# Interobserver variation of clinical oncologists compared to therapeutic radiographers (RTT) prostate contours on T2 weighted MRI

**DOI:** 10.1016/j.tipsro.2022.12.007

**Published:** 2022-12-30

**Authors:** Gillian Adair Smith, Alex Dunlop, Sophie E. Alexander, Helen Barnes, Francis Casey, Joan Chick, Ranga Gunapala, Trina Herbert, Rebekah Lawes, Sarah A. Mason, Adam Mitchell, Jonathan Mohajer, Julia Murray, Simeon Nill, Priyanka Patel, Angela Pathmanathan, Kobika Sritharan, Nora Sundahl, Rosalyne Westley, Alison C. Tree, Helen A. McNair

**Affiliations:** a The Royal Marsden NHS Foundation Trust, London, United Kingdom; b The Institute of Cancer Research/The Royal Marsden NHS Foundation Trust, London, United Kingdom; c Joint Department of Physics at the Royal Marsden and The Institute of Cancer Research, United Kingdom; d Clinical Trials and Statistic Unit, The Institute for Cancer Research, London, United Kingdom

**Keywords:** Prostate radiotherapy, MR-Linac, Radiographer contouring

## Abstract

The implementation of MRI-guided online adaptive radiotherapy has enabled extension of therapeutic radiographers’ roles to include contouring. An offline interobserver variability study compared five radiographers’ and five clinicians’ contours on 10 MRIs acquired on a MR-Linac from 10 patients. All contours were compared to a “gold standard” created from an average of clinicians’ contours. The median (range) DSC of radiographers’ and clinicians’ contours compared to the “gold standard” was 0.91 (0.86–0.96), and 0.93 (0.88–0.97) respectively illustrating non-inferiority of the radiographers’ contours to the clinicians. There was no significant difference in HD, MDA or volume size between the groups.

## Highlights

• The majority of radiographers prostate and seminal vesicle contours are within a range of clinicians’ contours.
• DSC of radiographer prostate and seminal vesicle contours are comparable to clinicians.
• There was no significant difference in the HD or MDA between the clinicians and radiographers when compared to a gold standard.

## Introduction

The implementation of online MRI-guided adaptive radiotherapy requires an increase in number of staff to be present at time of treatment compared to conventional radiotherapy [1], [2]. To utilise the staff effectively, some roles routinely performed by the clinical oncologist are being delegated to other staff members, for instance therapeutic radiographers (RTTs/radiographers) [3]. When evaluating contours outlined online on an MR-Linac, a comparison of the single online contour with a ‘gold standard’ or expert is undertaken. This process could include comparison with the expert contour performed offline or review offline of the online contour by the expert [4]. In either case the process is very dependent on the expert. Prior to undertaking online MR-Linac contouring, we evaluated the offline contouring of five clinicians and five radiographers, all of whom were experienced in MR-Linac treatment. The aim was to provide a baseline of an acceptable range of contours created by the clinicians to compare with radiographers. We hypothesized that if the radiographer contours were within the range of the clinician contours, then online contouring would be acceptable.

## Materials and methods

The interobserver variability of prostate and seminal vesicles (SV) contours was assessed offline. Five radiographers and five clinicians, with one to three years of MR-Linac experience, contoured the prostate and SV on a treatment planning system (Monaco treatment planning system, Version: 5.59.02 Research, Elekta, Stockholm, Sweden) on 10 T2-weighted MRIs acquired on an Elekta Unity MRL (Elekta, Stockholm, Sweden), from 10 patients. Two clinical target volumes (CTV) were created using each of the 100 contours:

High dose CTV: prostate plus 1 cm proximal SV.

Low dose CTV: prostate plus 2 cm proximal SV.

A simultaneous truth and performance level estimation (STAPLE) algorithm generated structures in ADMIRE (Version: Research 2.0 Elekta AB, Stockholm, Sweden) using the five clinicians’ contours to create “gold standard” high dose CTV and low dose CTV structures [5]. Fifty high dose CTV and fifty low dose CTV structures were created from both clinicians’ and radiographers’ contours. Each radiographer and clinician structure was then compared with the gold standard structure using Dice similarity coefficient (DSC), mean distance to agreement (MDA) and Hausdorff distance (HD). DSC is an overlap metric, such that 0 equals no overlap and 1 equals perfect overlap. MDA and HD are similarity metrics, with MDA measuring the mean distance comparable points on two surfaces would need to move to overlap and HD describing the maximum distance between two comparable points. Since we expect the radiographer contours to have slightly lower DSC than clinicians, because of the bias introduced by defining the gold standard as the average contour of the clinicians, we tested non-inferiority based on the radiographers being within 0.025 DSC of the clinicians. This corresponded to a reduction of the median clinicians’ contour from 0.93 to 0.905, a DSC threshold we previously determined to result in acceptable plans [6]. The mean distance to agreement (MDA) and Hausdorff distance (HD) were compared using a Mann Whitney *U* test. The volumes of radiographer and clinician contours were compared using a Mann Whitney *U* test.

## Results

**Fig. 1:**
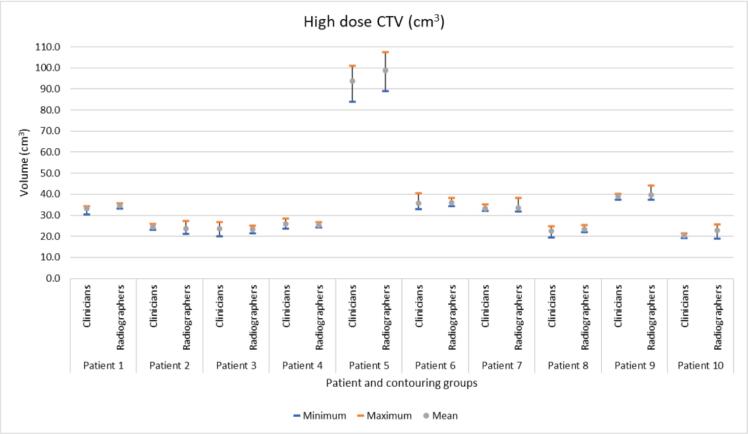

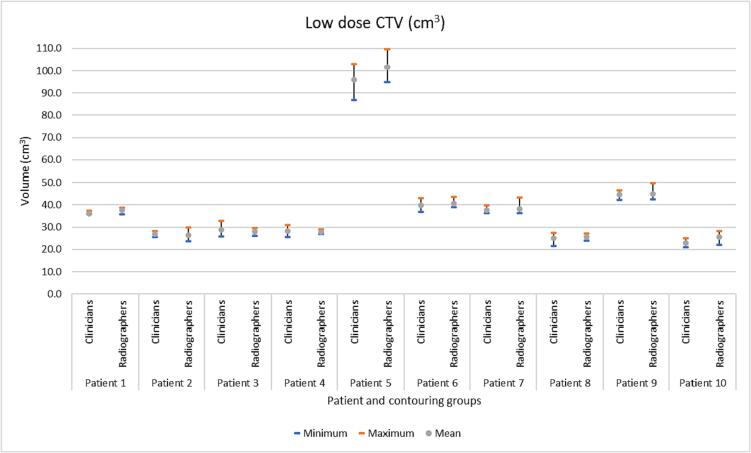
(a). Summary of 100 volumes for the radiographer and clinician contoured high dose CTV for ten patients’ images (five radiographer and five clinician contours per patient). Fig. 1b. Summary of 100 volumes for the radiographer and clinician contoured low dose CTV for ten patients’ images (five radiographer and five clinician contours per patient).

*High dose CTV;* Of the 50 high dose CTV radiographer structures, 30 volumes were within the clinicians’ volume range (Fig. 1a), six smaller (1% to 8%) and 14 larger (1% to 19%), and were not significantly different (p = 0.60). There was no significant difference in the HD or MDA, between the clinician and radiographers’ contours when compared to gold standard (*p* = 0.84 and *p* = 1 respectively) (Table 1). The DSC when comparing the radiographers’ contours to the gold standard was significantly different (lower) than the DSC from clinicians’ contours and gold standard (*p* = 0.018). However, the DSC from radiographers and gold standard was significantly higher than the DSC from the ‘clinicians and gold standard − 0.025′, indicating that the radiographers were non-inferior to the clinicians’ contours (*p* = 0.017).

**Table 1:** Dice similarity coefficient (DSC), mean distance to agreement (MDA) and Hausdorff distance (HD) for radiographer and clinician contours compared to the ‘gold standard’ STAPLE contour. Results presented as median (range).

	DSC		MDA (mm)		HD (mm)	
	**Clinicians**	**Radiographers**	**Clinicians**	**Radiographers**	**Clinicians**	**Radiographers**
High dose CTV	0.93 (0.88–0.97)	0.91 (0.86–0.96)	0.8 (0.4–1.9)	0.9 (0.5–1.6)	5.3 (3.2–9.9)	4.8 (2.9–9.5)
Low dose CTV	0.92 (0.86–0.97)	0.91 (0.83–0.96)	0.9 (0.8–1.0)	0.9 (0.4–1.5)	5.3 (3.2–10.0)	5.1 (3.3–12.2)

*Low dose CTV;* Of the 50 low dose CTV radiographer structures, 35 were within the clinicians’ volume range (Fig. 1b). Three were smaller (1% to 7.5%) and 12 were larger (1% to 13%) and were not significantly different (p = 0.67).

The radiographers’ DSC and gold standard were significantly higher than the DSC between the ‘clinicians and gold standard − 0.025′, indicating that the radiographers were non-inferior to the clinicians’ contours (*p* = 0.031). There was no significant difference in the HD or MDA between the clinicians and radiographers when compared to gold standard (*p* = 0.75 and *p* = 0.155 respectively).

**Fig. 2:**
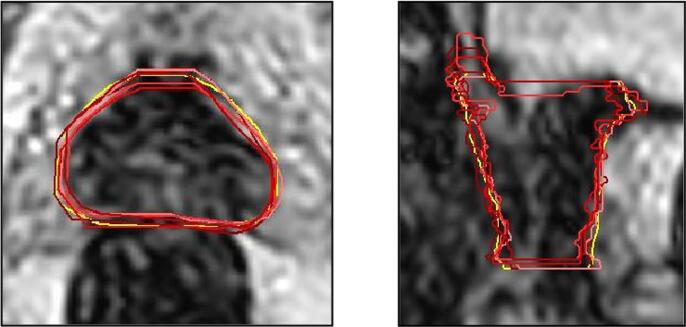

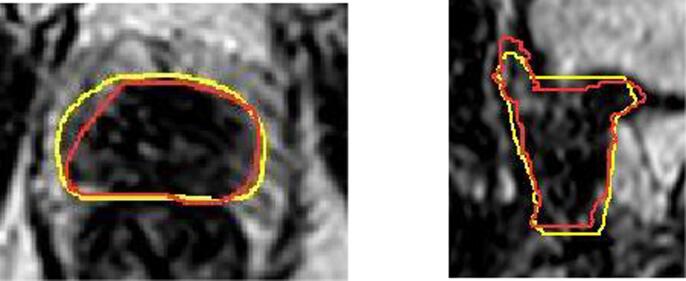
(a) Five radiographers’ contours (red) superimposed onto ‘gold standard' (yellow) contour illustrating discrepancies at base and apex. Median Dice similarity coefficient (DSC) = 0.95. Fig. 2b - Worst case: Radiographer contour (red) superimposed onto ‘gold standard‘ (yellow) contour illustrating discrepancies at base and apex DSC = 0.83.

On visual inspection (see Fig. 2), variations at the base and apex of CTV contributed to the largest difference in contour volume and the lowest DSC was 0.83 overall (low dose CTV).

## Discussion

Of the 200 high and low dose CTVs, 60% and 70% of the radiographer contours respectively were within the clinicians’ range. The contours which were smaller and would result in a target miss, hence of greater concern, were < 8% smaller, equivalent to 4 cm^3^ of the high dose CTV and 2.6 cm^3^ of the low dose CTV. The larger contours, although ensuring the target was encompassed, may increase the dose to organs at risk (OARs). Although statistically significant inter-clinician variability in prostate contour volumes were found in a study investigating inter-observer variation of five clinicians, the outcome with respect to the irradiated volume of the rectum and bladder was not clinically relevant [7]. The greatest agreement in contours at the prostate/bladder and prostate/rectum interfaces was thought to be the reason. Although we observed discrepancies at the prostate base as well as the apex, we found no significant difference in the volumes of the contours by five radiographers and five clinicians. It has been noted when comparing plans generated using gold standard contours versus auto segmented contours [8] or when comparing 25 observers contouring from one CT image [9] that a poor DSC did not necessarily result in poor dose coverage. When comparing plans generated using gold standard contours versus auto segmented contours the mean ± SD DSC was 0.87 ± 0.03, but the D98 ± 2% and V95% for prostate and its 3 mm expansion were within 2% (3 Gy) of each other, except for one case with a DSC of 0.82.

The variations at base and apex were the greatest and may have contributed to the significant difference found when comparing the DSC raw data (Fig. 2). There is an inherent bias towards the clinicians’ contours when using a gold standard created from the clinicians’ contours. By creating a threshold for an acceptable DSC of 0.905, a threshold we previously determined resulted in acceptable plans [6], we found that the DSC from the radiographers’ contours were significantly greater, illustrating good agreement. Contouring on MRI is known to result in less inter-observer variability than CT [10] and our DSC threshold of 0.905 is higher than studies using CT.

Delineation errors are recognised as a potential source of error in the conventional radiotherapy pathway [11]. Incorporating this task into the online workflow with multiple observers has the potential to increase this risk [12]. Training programmes have been shown to improve consistency but had not been completed by the radiographers in this study and may improve the results further [13]. The effect of the variations on dose to the target and OARs has not been established in this study and may not be clinically relevant, as in the Livesey study [7], however this needs to be established.

## Conclusion

The majority (>60%) of radiographers’ contours were within the range of clinicians’ contours. Although the DSC and gold standard comparisons between the two groups were significantly different there was no significant difference in other metrics used, including a non-inferiority test between DSC. Clinical relevance measures, for example dose, should be investigated alongside contour comparisons. Training programmes may improve agreement.

## Notes

The author(s) confirm that written informed consent has been obtained from the involved patient(s) or if appropriate from the parent, guardian, power of attorney of the involved patient(s); and, they have given approval for this information to be published in this case report (series).

## Declaration of Competing Interest

The authors declare the following financial interests/personal relationships which may be considered as potential competing interests: Dr Helen A McNair reports financial support was provided by National Institute for Health Research and Health Education England. Alison Tree, Angela Pathmanathan, Rosalyne Westley reports a relationship with Elekta Ltd. Alison Tree reports a relationship with Accuray Inc. Alison Tree reports a relationship with Varian Medical Systems Inc. Alison Tree, Sophie Alexander reports a relationship with Cancer Research UK that. Research at The Institute of Cancer Research is also supported by Cancer Research UK under Programme C33589/A28284 and C7224/A28724.
